# The Changing Landscape of Diverse HIV Treatment and Prevention Interventions: Experiences and Perceptions of Adolescents and Young Adults in Rural KwaZulu-Natal, South Africa

**DOI:** 10.3389/fpubh.2019.00336

**Published:** 2019-11-15

**Authors:** Thembelihle Zuma, Janet Seeley, Lindiwe O. Sibiya, Natsayi Chimbindi, Isolde Birdthistle, Lorraine Sherr, Maryam Shahmanesh

**Affiliations:** ^1^Africa Health Research Institute, Durban, South Africa; ^2^London School of Hygiene and Tropical Medicine, London, United Kingdom; ^3^University College London, Institute for Global Health, London, United Kingdom

**Keywords:** HIV interventions, HIV prevention landscape, adolescents, rural KwaZulu-Natal, South Africa

## Abstract

In sub-Saharan Africa, adolescents and young adults aged 15–24 years constitute 36% of an estimated 1. 3 million new HIV infections. Complex biological, social, behavioral and structural factors, as well as cultural norms contribute to whether and how young people perceive, are aware of and experience diverse HIV interventions. This qualitative study explored experiences and perceptions of intervention types among adolescents and young adults, and how different interventions could hinder or facilitate HIV treatment and prevention for adolescents and young adults in rural KwaZulu-Natal, South Africa. Data were collected as part of a DREAMS impact evaluation at the Africa Health Research Institute, KwaZulu-Natal between May 2017–January 2018. We used a combination of rapid community mapping and participant observation in four communities, 58 individual interviews, and 10 group discussions with 61 participants, conducted with both adolescent girls and young women and adolescent boys and young men. Thematic analysis focused on the changing HIV prevention landscape as experienced by adolescents and young adults. Participants reported a mix of new and old biomedical, behavioral, traditional, and locally-developed HIV prevention approaches. The appeal of the newer approaches depended on the extent to which they resonated with existing traditional and longstanding HIV prevention methods and the extent to which they engaged with adolescents and young adults' sexual experiences and with the social and structural factors including gender-related issues. These data demonstrate that in this context, newer methods and approaches can and should synergise with existing methods and beliefs. The HIV prevention landscape is evolving rapidly. Good community links and engagement offer an alternative support structure that could embrace both locally-developed approaches and traditional practices This structure could potentially support feasibility and acceptability of new and old HIV prevention approaches, without creating an impression that new approaches always need to replace the old.

## Introduction

Despite developments achieved with HIV treatment and prevention efforts, the number of people who acquire HIV each year remains unacceptably high (1), particularly among young women and men (2, 3) who represent 27% of the world's population. In 2015, sub-Saharan Africa (SSA), had an estimated 1.3 million new infections among adults aged ≥15 years (4). Young people aged 15–24 years constitute 36% of these (4). Generally, in countries classified as having generalized HIV epidemics, adolescent girls, and young women (AGYW) acquire HIV 5–7 years earlier than adolescent boys and young men (ABYM) (2, 5).

Complex biological, social, behavioral and structural factors as well as cultural norms contribute to the extent to which adolescents and young adults perceive, are aware of and experience diverse HIV interventions (6–8). A review focusing on barriers to adolescent HIV care in sub-Saharan Africa identified fear of stigma, family reaction, fear of the impact of a positive HIV diagnosis, perceived risk with respect to sexual exposure and poor attitude toward younger clinic-attendees by healthcare providers as major barriers that continue to impact effective HIV treatment and prevention efforts among adolescents (9). Findings from other research show the impact of gendered social norms, family disruption, and entrenched social and economic inequalities on HIV risk among young adults (10–15). A review conducted by the World Health Organization (WHO), indicates that adolescents, including those infected by HIV, are particularly sensitive to the stigma associated with HIV and thus appreciate health care services that offer privacy and confidentiality (16). Other major facilitators identified in that review include support—from providers, caregivers, peers, and the community—and skills development to increase self-confidence, self-efficacy, and empowerment (16).

This ongoing challenge to reduce new HIV infections, and deliver timely diagnosis and successful HIV treatment and care among adolescents and young adults (3, 17, 18) has highlighted the need for combining different interventions. One such combination intervention is the DREAMS (Determined, Resilient, Empowered, AIDS-Free, Mentored, and Safe) Partnership, a broad combination of evidence-based health, educational, and social interventions, specifically designed to prevent HIV in AGYW (and their male sexual partners). The DREAMS Partnership is an investment by the U.S. Government President's Emergency Program for AIDS Relief (PEPFAR) office, Bill and Melinda Gates Foundation, Girl Effect (formerly the Nike Foundation), and other private sector partners, announced in 2014 and implemented in South Africa between April 2016–September 2018 (19). One of the five DREAMS intervention sites in South Africa is a high HIV prevalence and incidence area in rural KwaZulu-Natal in uMkhanyakude district (20).

Effectiveness of complex combination HIV prevention interventions, such as DREAMS will depend on population wide uptake and retention by those targeted (in this case AGYW and their male partners) (21, 22). We hypothesize that this will depend on how the different intervention types are perceived, experienced, and navigated by adolescents and young adults, in the interplay with community norms, social dynamics -in particular gender and intergenerational dynamics- and the evolving HIV prevention landscape (23). This study explores experiences and perceptions of intervention types among adolescents and young adults, and how different interventions could hinder or facilitate HIV treatment and care for adolescents and young adults in rural KwaZulu-Natal, South Africa.

## Methods

### Study Design

This study used a qualitative research design, combining rapid community mapping (24, 25), focus group discussions (FGDs) (26, 27), semi structured in-depth interviews (IDIs) with AGYW and ABYM (28) and participant observation, in order to understand complex social interactions and culturally informed norms which influence perceptions, experiences and how the changing HIV landscape is navigated by adolescents and young adults (29). Data reported in this study were collected between May 2017-January 2018, the first of 3 years of the DREAMS combination intervention in uMkhanyakude.

### Setting

The study included AGYW and ABYM living in Mtubatuba, one of the two sub-districts in uMkhanyakude that has had HIV combination prevention rolled out through the DREAMS partnership (30). The overall aim of DREAMS is to reduce HIV incidence in AGYW, through a combination of interventions that target community, family, male partners, and AGYW to promote safer sexual relations, social protection, biological protection, and empower AGYW (31). In the uMkhanyakude district, DREAMS is implemented in 2 sub-districts: Mtubatuba and Big Five Hlabisa. Within the district, the DREAMS core package, aimed at addressing HIV risk behaviors, HIV transmission, socio-economic vulnerabilities, and gender-based violence is delivered by five main implementing partners (IPs) and five sub-contracted community based organizations who work closely with government departments including the Department of Health, Social Development, and Basic Education, as well as the local municipality (22).

HIV prevalence in the Hlabisa sub-district is 24% among adults aged 15–49 years (32). The area is predominately rural and poor, with two thirds of households receiving social grants (33, 34) and ~92% of the population speak isiZulu as a first language. A fuller description of DREAMS core package and IPs delivering the intervention is provided elsewhere (19).

### Sample

**Figure 1:**
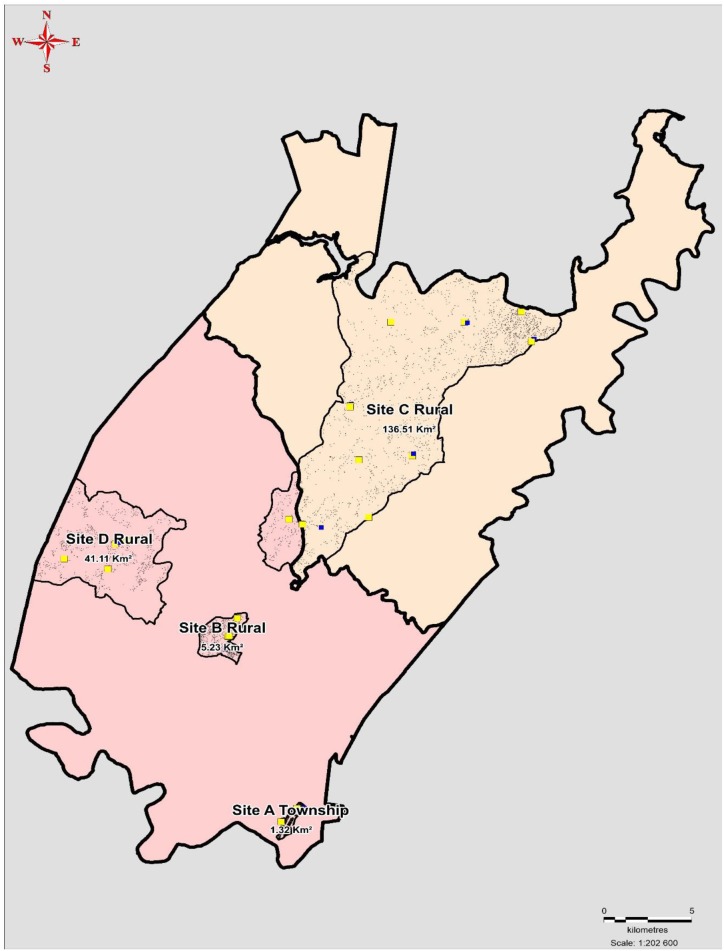
Local communities where the study was conducted.

Stratified purposive sampling was used to recruit AGYW and ABYM targeted by DREAMS between the ages 10–35 from four communities: 1 peri-urban township characterized by a high density of households (33), and three rural communities made up of scattered settlements as shown in Figure 1 (35). Within these communities there are eight secondary schools, 20 primary schools, and each community has one primary health care clinic. Participants were identified and recruited if: they resided in one of the four pre-identified communities, were within the age targeted by DREAMS, were willing to commit to the 24-month period prescheduled for data collection and provided written informed consent to participate in audio-recorded IDIs or FGDs. Written, parental/guardian informed consent was sought for participants younger than 18 years.

### Data Collection and Management

FGDs, IDIs, and community mapping were conducted by a trained team of eight social science research assistants (four females and four males) who were all native isiZulu speakers, the first language of the participants. Interviews were conducted in participants homes or venues where participants' felt comfortable such as schools, taking between 30 and 60 min. FGDs were held in community venues arranged with assistance of the local headman and in natural settings where groups meeting the study's inclusion criteria gathered and they took between 60 and 120 min. Separate male and female FDGs were held to maximize participant's ability to voice opinions, particularly on gender-related matters and interventions targeted specifically to males or females. Data collection tools were developed and piloted in two rural communities for a period of 2 months. At the end of IDIs and FGDs, participants were provided with a light snack. IDI and FGD guides consisted of open-ended questions presented in Table 1. Data were triangulated using FGDs, IDIs and community mapping to give more insight into the perceptions of participants and to complement their narratives as well as to recognize inconsistencies in the data. While FGDs addressed perceptions and general views, IDIs also sought personal experience of participants who were able to share them during the interview. Data collected during community mapping included observing activities and movement of different age and gender groups in order to quickly gain a broad understanding of the social context for adolescents and young people and the reach and coverage of different health services for sexual and reproductive health and places to manage chronic diseases. Recorded FGDs and IDIs were transcribed verbatim and translated from isiZulu to English by the same research team who collected the data. Translations were validated by the first author (TZ) who is a native isiZulu speaker, to ensure completeness and accuracy.

**Table 1:** Main topics addressed during IDIs, group discussions, and activities observed during community mapping.

**Approach**	**Topics**	**Probes**
Individual interviews	Experience and expectations of health care	Explore health care facilities used over the past year Explore experiences in health care settings, looking at barriers, and facilitators
	Experience and expectation of HIV testing	Ask if participant has ever tested for HIV and probe about their experience and the facility they used If participant has never tested probe about reasons Explore perceptions of HIV risk (as well as risk in the general community)
	Experiences with the wider DREAMS and DREAMS type interventions	Knowledge and exposure to DREAMS and other HIV prevention interventions Barriers and facilitators to what works in practice
	Parents and/or guardians involvement or support for DREAMS and DREAMS type interventions	Explore why/why not parents are involved Explore how this affects access and utilization of care
Focus group discussions	Experience and expectation of reproductive health and sexual health care	Perceptions of HIV risk in the community
	Experiences with the wider DREAMS and DREAMS type interventions	Knowledge and exposure to HIV prevention interventions including DREAMS (and DREAMS-like) interventions Experiences and perceptions of DREAMS (and DREAMS-like) interventions/activities
	Parents and/or guardians involvement or support for DREAMS and DREAMS type interventions	Explore why/why not parents are involved Explore how this affects access and utilization of care
Community mapping	Activities and movement of different age and gender groups	Reach and coverage of HIV prevention interventions including DREAMS (and DREAMS-like) interventions

### Ethics

Ethical approval was granted by the University of KwaZulu-Natal's Biomedical Research Ethics Committee (BREC) (Ref: BFC339/16) and the London School of Hygiene & Tropical Medicine's Research Ethics Committee (Ref: 11835) as well as the Hlabisa District Hospital, and the AHRI Somkhele Community Advisory Board. All the participants were asked for consent to participate in the study and to audio-record group discussions and individual interviews.

### Data Analysis

Thematic analysis was used to explore the changing HIV prevention landscape as experienced by AGYW and ABYM (36, 37). Data providing insights into the complex interplay between individual, interpersonal, and structural factors influencing participants experiences and engagement with HIV prevention were a particular focus of the analysis. Individual interview and group discussion data were organized and coded in NVIVO version 11. Initially, TZ and LOS used a tentative conceptual framework to approach preliminary analysis. The framework used questions addressed in IDI and FGD guides to code specific data related to: (a) AGYW and ABYM's narratives on HIV prevention interventions and sexual and reproductive health; (b) experiences with HIV prevention interventions; (c) navigating multiple and changing HIV prevention interventions; and (d) attitudes and perceptions of HIV prevention interventions. In addition, transcripts were coded to allow for descriptions of how AGYW and ABYM made sense and interpreted their daily lives in relation to HIV prevention and sexual and reproductive health care. Local knowledge was used to define and make sense of participant's lives. The research team examined the codes across and between IDIs and group discussions. Collated data were summarized into potential themes. Initial coding was then followed by the team's joint repeated review of thematic categories which were defined and refined throughout the analysis process according to Braun and Clarke's thematic analysis steps (36). The research team recognized how age, beliefs, personal experiences, and being part (or not) of the same community as the research participants may have impacted the nature and outcome of the findings. The team conducted regular debriefing meetings to address their experiences and how they related to the participants and the context. The process provided an opportunity to identify and address assumptions and preconceptions during data collection and analysis, as well as explore emerging themes with the team. Reflexive iteration provided a platform to visit and revisit the data as it was collected in order to connect it to the emerging insights. This iterative process led to refining the focus and understanding of data and linking it back to the research questions and objectives. Preliminary analyst triangulation was undertaken by TZ, JS, and MS who iteratively reviewed the initial themes, reconciled differences and discrepancies, and refined themes throughout the analysis process. Trustworthiness of the data was ensured through reviewing field notes, debrief sessions, and peer reflections between the field team and authors. Credibility of the data was demonstrated by providing verbatim quotes from participants narratives.

### Findings

#### Participants Profile

**Figure 2:**
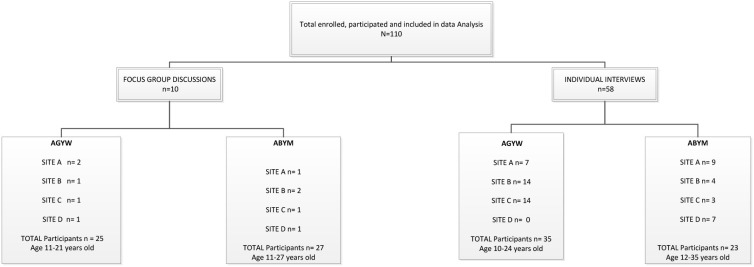
Participants profile.

Primary data for this study were drawn from 110 participants as shown in Figure 2. IDIs included 58 participants with AGYW and ABYM. Of the 58 IDIs, 35 were conducted with AGYM aged 10–24 years, 22 of whom were in high school (grade 9–11); 10 in primary school (grade 4–6); and three were out of school (completed secondary level education—grade 12. Additionally, the study included 23 IDIs with ABYM, eight of whom were in high school (grade 10–12); one in primary school (grade 7); and 13 were out of school (completed matric). In addition, 26 AGYW participated in 5 FGDs spread over the four sites and a total of 35 ABYM participated in five group discussions. Group discussion participants were aged between 13 and 26 years.

**Figure 3:**
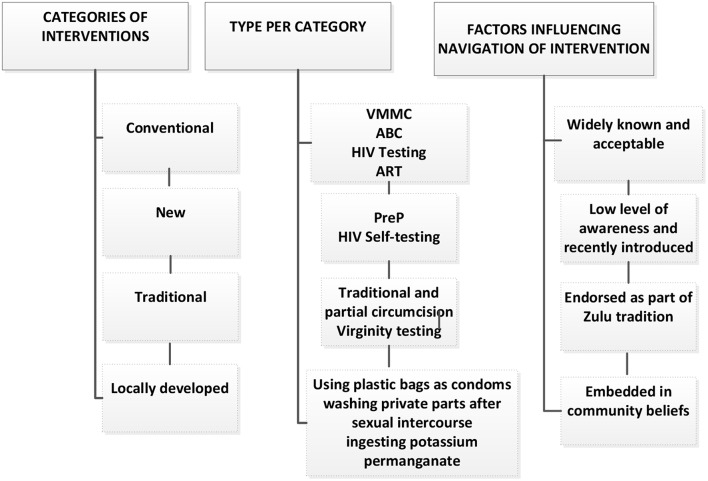
Diverse HIV prevention interventions.

Participants described interventions according to how long they had been known, how they were experienced and what they had heard about them in their communities. In both IDIs and group discussions, participants reported different categories of interventions including biomedical, behavioral, traditional, and locally-developed interventions, highlighted in Figure 3. Broad categories that emerged were related to information and knowledge of interventions, exposure to and endorsement of interventions as well as diverse sexual dynamics shaped by particular social and cultural experiences. Participants' information and knowledge influenced how interventions were perceived and experienced. Additionally, duration of, exposure to and endorsement of interventions influenced how they were navigated.

### Conventional Interventions

Conventional interventions refers to well-known and acceptable interventions which according to participants, were to some extent available in their communities and public health care facilities. These interventions included: medical male circumcision (VMMC), abstinence, being faithful, condom use (ABC), HIV testing and treatment. Participants reported that these longstanding interventions were either less available or less accessible than participants would have wanted them to be and as such, they were described as not always contributing to their choice.

Additionally, the understanding of the protection afforded by some interventions, led some not to seek other prevention methods. For example, most of the male participants in the study reported undertaking circumcision when they were between age 13 and 18, an intervention they thought was “protective and helped to prevent HIV transmission.”

“….and I myself had slept with an HIV positive girl and found out that she is positive after the intercourse, but I didn't catch it (HIV) because of circumcision. If you have slept with an HIV positive person several times without a condom and still be HIV negative, what will you say protected you, circumcision works…: SITE A IDI, Male, out of school

VMMC provided an opportunity for welcomed sexual encounters and experimentation which made male participants “happy” since they did not use condoms for protection against STIs to enhance sexual pleasure. They further reported that they “found endurance during sexual encounters” which brought satisfaction to their female partners, however they expressed concerns around performing oral sex, which they perceived put them at risk even though they were circumcised. On the other hand, female participants reported that VMMC was available in local primary health care clinics and offered by unknown organizations. However, no accounts of sexual satisfaction related to VMMC were reported by this group. Popularity of VMMC was supported by its connection to traditional beliefs, understanding that it reduced HIV transmission, sexual virility and its endorsement by the King of the Zulu nation, as shown in other studies (38, 39).

“For me I heard about circumcision at a very early age, I did not care about it, I said I would not get circumcised, as years went pass I had the feeling of liking it, later on when they asked for young men and explained that it minimizes the chances of transmission of HIV, I realized that maybe if I do not use a condom, rather I get circumcised”: Site D FGD, young men

Some participants had not undergone VMMC and they reported hoping to do so in the future. Reasons for not having done VMMC included fear of testing for HIV, as they understood that testing HIV negative is a requirement for a community-based VMMC procedure. Participants reported that if they tested positive, they would have inadvertently disclosed their status as VMMC was mostly conducted in local community halls where young men gathered to get the procedure done. According to South African National guidelines for circumcision, all men requesting circumcision for prevention should be offered an HIV test and appropriate post-test counseling to ensure that more men in the community know their HIV status and are better able to take care of themselves, either to remain HIV free, or to take ART that will slow the progression to AIDS. However, the guidelines make it clear that the test is not a prerequisite for circumcision (40). Due to the general belief among participants, and seemingly the general community that an HIV test is required, an opportunity is missed for linkage or identification of men who are lost to follow up. For some, fear that they might test HIV-positive outweighed engaging with HIV-related health care.

Other types of longstanding interventions included behavioral interventions such as ABC. Information and knowledge about these approaches was obtained from health care facilities, schools, and on the media. Both male and female participants reported that condom use information was obtained during curriculum-based interventions and community-based condom distribution provided by DREAMS implementing partners. Participants said that the ABC strategy protected against infection from STIs and unwanted pregnancy. Abstinence and being faithful were reported as uncommon prevention strategies and mostly used by young girls who went to study away, sent by parents to be at boarding school and those who practiced virginity testing because they delayed starting sexual relationships. There was a perception that these two approaches were not easy to adhere to as most young people in the community started having relationships and experimented with sex when they reached secondary school. Sexuality was generally discussed among peers and participants felt that this created sexual knowledge gaps because as young people they were inexperienced and did not have much information.

“The information is scarce. This information we have is that which we get when we talk to each other”: SITE D FGD, young men

Generally, participants discussed that their peers must have stopped following these behavioral interventions as they were perceiving an increase in pregnancy rates among AGYW. According to Bhana (41), the tension experienced by adolescents and young adults is on the one hand due to their developing sexuality which involves experimenting sexually, and on another hand the responsibility they have not to contract STIs which are a sign of poor morals and an inability to take care of themselves (41). Young men believed that condoms were uncomfortable and boring, therefore they were not used often, even though they knew they had a responsibility to use them for protection.

“Oh, sometimes you find that if you are using a condom there is some air that gets inside her so that is why she don't like it”: SITE C, FGD, young men

Participants mentioned that over the past few years, condoms started being delivered in local food trading facilities within their communities by different organizations. The research team observed that, condom distribution and demonstration were conducted for individuals and groups and distributed on a weekly basis in informal food trading facilities by DREAMS implementing partners. However, participants discussed that community delivery of condoms was stigmatized as it could not be discrete, particularly for AGYW.

Both male and female participants reported that AGYW were fearful of collecting condoms in public places in case they were seen and judged, unlike their male peers. Conservative social norms were perpetuated by parents who believed that these interventions condoned immorality and infidelity in AGYW. It was considered taboo to have women take responsibility of their sexuality. Intergenerational communication around the inappropriateness of women who exercised their right to sex was persistent and common. In both IDIs and group discussions, it was acknowledged that AGYW had less power to negotiate condom use which could be the reason for the increasing pregnancy rates witnessed in the community. Power relations operating between men and women further constrained AGYW to make decisions around their sexuality and practice safer sex. Female participants reported that in some relationships, even if a sexual partner promised to use a condom, it was removed during intercourse without consent which created anxiety over thoughts of pregnancy or infection with an STI.

These sexual norms, expectations, and structural factors limited AGYW to access and use ABC, and as such increased their risk and vulnerability. Bhana (41) argues that the exercise of male power enhances heterosexual masculinity but compromises girls' well-being and makes them vulnerable to sexual risk as the responsibility for sexual relationships is placed on them (41). Even though ABC was widely known, and condom distribution had improved with the introduction of DREAMS, young people were ambivalent about the usefulness of ABC. This finding corroborates the work of Wickstrom who associates the low uptake to the loss of sexual intimacy (42). In the current study, young men were less likely to report using condoms and did not perceive themselves as at risk, they believed that “a sweet is not nice inside its wrapping paper”: Site B FGD, young men. Condomless sex with regular partners was not considered risky even when a partners HIV status was unknown, a phenomenon that has been reported elsewhere (43, 44).

HIV testing was reported as a common HIV prevention strategy. Participants said that HIV testing in both health care facilities and within communities promoted awareness and easy access, particularly for young people who often found it challenging to use health care facilities. About half of both male and female participants in IDIs reported having been motivated to test for HIV in the past year, as a result of an increase in community-based HIV testing services which they said were more private and confidential.

It was acknowledged that HIV treatment was widely available in local primary health care facilities. However, there was a general feeling that being seen at an “HIV clinic” was embarrassing and it made young people feel ashamed about being HIV-positive at a young age. While female participants said it exposed the fact that they had had sex, male participants felt that they would not be well-received in health care facilities by staff and older community members. Those who said they will initiate ART said they had support from their family, specifically their parents. Even though all participants believed that there were benefits in taking ART and initiating early, they did not know about the benefits of ART on onward HIV transmission. Some participants inquired whether “doctors” had approved the fact that when ART is adhered to and initiated early, it reduced transmission. They said that if more people in their community knew that ART could prevent HIV transmission, those who were HIV-positive would adhere and protect their partners.

“HIV positive people can adhere to their treatment and won't infect others”: SITE A, female, in school.

### Ambivalence Around New HIV Information and Interventions

Participants shared contradictory ideas about HIV interventions recently introduced in their communities. Contributing to the uncertainty held about such interventions was the low level of awareness and information held by participants. It was reported that interventions such as HIV Pre-Exposure Prophylaxis (PreP) “a pill that you take if you are HIV negative” and HIV self-testing (HIVST) were new, and all participants lacked information about these interventions. Information and knowledge related in IDIs and FGDs was based on what participants had heard and not on personal experiences as these two interventions were not available to them or in the local primary health care facilities.

In individual interviews, some participants mentioned that PreP was only available for women who sell sex, provided by a DREAMS implementing partner. Some participants confused it with post-exposure prophylaxis (PeP) which they knew was available in primary health care facilities. Concerns expressed by participants about the use of PreP by young people included fearing that young people would stop to use condoms and acquire other STIs, side effects related to the use of PreP and the potential discrimination that could be directed to those who used PreP, especially if they were young females and if PreP was accessed from health care facilities where they said they may experience HIV-related stigma, associated with being seen at an “HIV clinic.” On the one hand, participants welcomed the idea of a tablet that would prevent HIV and expressed that they wished it was available. On the other hand, participants communicated concerns around the longer-term effect on dis-inhibiting young people's sexual behavior. These findings concur with the need for clear education and messaging to accurately explain the effectiveness of PrEP (45), and the lack of an association between PrEP use and changes in sexual risk behavior (46), in contrast with the ambivalence that is suggested by young participants in this study:

“Yes, I think this pill [PreP] is good and it's going to help so much, most especially here in young people it will help so much. Though, as much as it will be of much help and have a good impact in reducing HIV among young people, but again what I see is that young people will continue even more with unprotected sex. Since they would know, what would they use condoms for if they know that they will not be infected, you see…”: Site D IDI, 19-year-old male, out of school

A similar circumspect enthusiasm for HIVST was reported. A predominant perception about HIVST was that it could help to mitigate HIV-related stigma experienced by individuals testing in health care facilities and improve HIV testing rates among young men who generally expressed less desire to test for HIV in health care facilities. Participants also said that self-testing could reduce traveling costs and time spent at health care facilities as the self-test could be done anywhere the individual felt comfortable. However, male and female participants were concerned that HIVST could lead to emotional distress and suicide, denial of an HIV positive status and a reduction of linkage to care by individuals who tested on their own without receiving HIV counseling by a trained counselor to prepare them for their results.

“It [self-testing] will help a lot because when you are testing at the clinic, most of the time you have those moments where you are afraid that the person testing you will see your results and even worse because they are from the same community as you….”: Site B IDI, 22-year-old female, out of school

Similar barriers and facilitators of HIVST were indicated by key stakeholders involved in HIV treatment, care and HIV counseling and testing (HCT) policy in a study conducted in South Africa (38). Another study conducted with adolescents in Cape Town, South Africa, showed a high degree of fidelity and acceptability of HIVST (47). In the current study, a few participants did not feel there was any difference between testing at a health care facility and self-testing because at the end of the day—“one needed to go to a health care facility to access treatment if they were HIV-positive.” However, these views were based on anticipated use as both PreP and HIVST were not available in these communities, therefore what could happen in participants lives when faced with real options could be different.

An additional intervention reported by participants was a behavioral curriculum-based intervention provided by DREAMS implementing partners targeting AGYW. This intervention was described as a new strategy to prevent HIV. The curriculum based behavioral intervention delivered through DREAMS was mostly reported by AGYW who were in school and had been part of such an intervention. Most ABYM and out of school participants did not know this intervention and did not participate in the intervention.

“I think it is a good programme because there are kids that are doing grade 8. They get into love relationships with grade 12 boys and brag about it but after receiving the sessions they start knowing that boys would only want to play with them. I think they are good trainers. But, from my own view I think the programme is teaching only girls and they don't include boys, but we are all teenagers and we all make mistakes, why are they excluded? We will all die of HIV because when we are being trained on how to abstain, we will be dating those boys who are not part of it” Site B IDI, 22-year-old female, in school

Exclusion of men from the widespread curriculum-based education introduced through DREAMS, was considered to be counter-productive and inequitable, by both AGYW and ABYM. Participants felt that this intervention limited impact by leaving out ABYM and therefore did not play a major role in reducing HIV incidence among AGYW.

### Traditional Practices

Traditional practices were described as HIV prevention approaches commonly conducted as part of Zulu tradition which included traditional male circumcision, partial male circumcision, and virginity testing. Traditional circumcision is performed in a non-clinical setting carried out for cultural reasons, mainly among adolescents and young men aged 13–20 years (48). This practice is viewed as a sacred and indispensable cultural rite intended to prepare young men for the responsibilities of adulthood among different South African tribes (49). Inconsistent messages around traditional male circumcision and HIV transmission were reported by male participants. While some male participants said that it protected men from acquiring STIs, including reducing HIV acquisition, others said, “traditional male circumcision prevented HIV transmission, but not other STIs” and only reduced HIV transmission by 60% and therefore, not fully effective. Though traditional male circumcision was a popular traditional practice among ABYM, it was reported that VMMC was better because it was done medically and within a clinical setting and female participants did not report on this HIV prevention strategy. Male participants reported that they had heard that a lot of males had been injured and sexually impaired through circumcision performed as part of boys' rite of passage. Experiences of medical complications requiring treatment after traditional male circumcision included septicaemia, gangrene, severe dehydration, and genital mutilation had been reported, particularly among the Xhosa tribe (50, 51).

“one of the reasons is that there is an incident that if you are being circumcised in the place called “mountain” (a sacred place where young boys' rite of passage is performed), there is a possibility that there would be a fault in your circumcision, you get hurt in your private part and then you end up being unable to have sex. You end up being a paralyzed individual”: Site B FGD, young men

In group discussions with ABYM, partial circumcision *(ukuqhatha*) (52), where the foreskin is not removed, but an elastic band of tissue under the penis gland is cut, allowing the foreskin to move easily back and forth was also reported. Participants held different understandings of partial circumcision in the context of HIV prevention. Even though some ABYM said that it was done as part of HIV prevention, others said that it was an old tradition done by young men to help to avoid sensitivity and pain during sexual intercourse, as the external foreskin remains intact. None of the participants reported having undergone partial circumcision.

Male and female participants also reported virginity testing as a cultural practice and an HIV prevention approach which was regarded as a vital social tool to bring pride to the virgin girl, the parents and the community (42). The process of virginity testing involves undergoing physical examination by older women in the community to find out whether the girls' hymen is intact (53). The practice came to restoration in recent years in most areas of KwaZulu-Natal to fight against teenage pregnancies and HIV. In both interviews and group discussions, virginity testing was met with a variety of views. Some AGYW and ABYM considered the practice to be valuable. They said that older women taught AGYW about sex and sexuality and how a young girl ought to behave.

“Then there are also organizations that teach girls although I do not know them by their name but there is one that teaches girls at X High School after school hours. The other thing that helps in trying to protect young girls from HIV is the availability of mothers who perform virginity testing within the community. They test them for virginity and those girls attend the reed ceremony. Ehh according to my thinking it prevents HIV because when she behaves in a proper way it is when she cannot get HIV”: Site B FGD, young men

However, most of the participants, including both AGYW and ABYM said that the practice of virginity testing was no longer relevant as an HIV prevention approach, placed the burden on women and girls to behave within the cultural space and was seen as encroaching on young women's private lives as also reported by Scorgie (54).

“I think that virginity testing is not a good thing, it has disadvantages because sometimes when you did not go for virginity testing maybe because you don't have money, people in the community will misjudge you about not being a virgin or if you do not have beautiful breasts, they will say that you are sleeping around with boys,”: Site B IDI, 17 year old female, in school

Out of the eight AGYW who spoke about virginity testing in IDIs, half had participated in the practice and reported that life skills integrated into the practice taught them about the benefits of delaying pregnancy and finishing school. However, participants who did not endorse the practice further said, the pricing of virginity as a practice that defined the integrity of women further condoned women's rights to sex. Some participants reported that the practice was merely done for parents or as a result of social pressure and not for the AGYW themselves, as a result, most fell pregnant as soon as they left their homes to start tertiary education. Four of those who had undergone virginity testing reported that the practice was important to them and they had not done it out of pressure, but it was done so that they could be examples to other young girls. However, the same was not expected from young boys.

Traditional Zulu sexual and reproductive health practices (male circumcision and virginity testing) were ubiquitous and had taken on new signification for young people in the context of the HIV epidemic. Acceptability and experiences of these interventions were variable, with a suggestion that virginity testing in particular could marginalize and stigmatize young girls' sexuality further as also suggested in other studies (55, 56). Conflicting messages around controlling AGYW's sexual behavior and endorsement of virginity testing perpetuated unequal power dynamic between young women and men.

### Locally-Developed Approaches

Locally-developed approaches, stemming from embedded community beliefs included using plastic bags as condoms, washing private parts after sexual intercourse, ingesting potassium permanganate or drinking a mixture of coca cola and disprin (aspirin) to kill HIV in the blood. These approaches were mostly reported by male participants in the rural sites, with participants in the more urban sites expressing skepticism about these beliefs. For example, one male participant in an IDI in the semi-urban site said that he did not think washing a private part after sexual intercourse was an effecting HIV prevention approach though he had heard such reports in his community.

Locally-developed practices were reported in sites where HIV prevention and sexual and reproductive health services were difficult to access due to distance. Generally it took >1 h to travel to local health care facilities and about 60.8% community members walked to facilities while 38.8% used public transportation and 0.4% used their private transport (57). We found that myths and misinformation about HIV and reproductive health prevailed in areas that were located far from health care facilities and they were much more common among AGYW and ABYM.

“It is for a girl to drink a mixture of coke and disprin after having sexual intercourse, so that the sperms will not penetrate through, but remain on the surface. It prevents pregnancy, but mainly the transmission of HIV. Another method is consuming the seed of dagga (marijuana); the girl drinks the seed of dagga and it becomes a permanent contraceptive. It is taken as you would be drinking a cold drink or water, it is said when taken, it goes and blocks something in the uterus”: Site C IDI, 19-year-old male, in school

Factors relating to information and knowledge of HIV prevention as well as cultural and structural factors impacted how adolescents and young adults perceived and navigated health care services.

It is this other thing that guys do each time they were having an unprotected sex where they will rush to a tap right after sex and open the tap [laughing] and it is well-known to work: SITE A, IDI, Male, out of school

A variety of alternative approaches, mentioned in the quotations above are used by young men in the absence of effective, trusted, and known measures to protect themselves from HIV. These alternative approaches were also notably used in the absence of access to services. In the most rural areas where we found a lack of HIV-related information and where it was difficult for young men to access health care services, young men still made means to protect themselves.

## Conclusions

This study demonstrated that there is high individual awareness of some biomedical HIV-related interventions such as condoms, HIV testing, and VMMC. Some participants mentioned having used these services and others reported knowing where to access them. Community delivery through DREAMS was popular, particularly among in school AGYW as it overcame the lack of privacy and inconvenience of accessing interventions in the facility and there was cautious enthusiasm for the newer interventions such as PreP and HIVST. However, the traction of the newer approaches depended to the extent to which they resonated with the changing social and cultural norms as well as the extent to which men were also engaged, for example VMMC. It is, thus, important to identify when and how new approaches build onto existing ones. In addition, it is important to provide supportive and enabling individual and community educational packages and services that are culturally sensitive and open to rural contexts for new intervention approaches to be accessible, understandable, acceptable, and applicable. Making these interventions widely available could also erode persisting incorrect HIV-related messages, particularly around HIV transmission and treatment.

The study findings indicate that there is tension experienced by adolescents and young adults as they navigate HIV prevention interventions and their community (both gender and intergenerational) sexual norms to evolve and experience their own sexuality. There is, therefore, a need to increase access to knowledge, social support, and provide safe networks for young people in order to improve access and utilization of HIV prevention interventions. New interventions need to embed the prevention interventions within the existing narratives of sex and sexuality. Good community links and engagement could offer a supportive structure that could handle social and culturally bound issues and facilitate introduction of newer HIV prevention approaches, without creating an impression that new approaches are meant to replace the old. Acknowledging young people's context, existing and changing traditional practices and tailoring interventions and messages to these is key to successful implementation. Combining diverse HIV prevention approaches could be feasible and acceptable when resources to support interventions are adequately provided.

## Implications of Evidence and Way Forward

Understanding the context into which new interventions are implemented and engaging young people around their perceptions and understanding of new interventions will help improve knowledge, navigation and clear their worries and concerns. For example, in the case of HIVST, what does this mean for young people who think that linkage to care will be a challenge after an HIV positive diagnosis? How can these technologies be used to support young men? How can integrating HIV testing modalities with the knowledge around HIV treatment reducing HIV transmission support engagement with care? In the case of PreP, how will stigma and side effects related issues be dealt with to motivate utilization? To what extent can PrEP overcome the barriers to condom uptake and use? What can we learn from teenage pregnancies and the failure to implement contraception before first sex in the roll out of PrEP? How can young men and women use these old and new technologies to navigate sex in the context of existing conservative sexual norms complicated by the fear of HIV?

## Strengths and Limitations

Participants in FGDs resided in the same communities and knew each other, this could have potentially led to social desirability bias. We acknowledge that in IDIs some participants did not share their own personal experiences and rather reported on perceptions and general views. To try and illuminate these restrictions, we used IDIs, FGDs, and community mapping methods in different sites, thus confirming findings from different independent sources. We acknowledge that data were not collected on some important factors likely to shape the navigation of health care, such as the decrease in use of ABC and participants' exposure to different interventions, more research is needed to understand these factors. More research is also needed to understand who the guardians of sexual knowledge in this setting are and how sexual knowledge is transferred from one generation to another as well as how it builds up as new interventions become part of the prevention landscape.

## Data Availability Statement

The datasets generated for this study are available on request to the Africa Health research Institute through the corresponding author.

## Ethics Statement

Ethical approval was granted by the University of KwaZulu-Natal's Biomedical Research Ethics Committee (BREC) (Ref: BFC339/16) and the London School of Hygiene & Tropical Medicine's Research Ethics Committee (Ref: 11835) as well as the Hlabisa District Hospital, and the AHRI Somkhele Community Advisory Board. All the participants were asked for consent to participate in the study and to audio-record group discussions and individual interviews.

## Author Contributions

TZ contributed toward development of the tools, collected and analyzed the data, and wrote the first draft. LOS collected data and contributed toward initial data analysis. NC, IB, and LS contributed to data analysis and revised the manuscript. JS contributed to the study design, analyzed the data, and revised the manuscript. MS is the PI of the study and contributed to the study design, data analysis and oversaw writing of the first draft, and revised the manuscript. All authors read and approved the final manuscript.

### Conflict of Interest

The authors declare that the research was conducted in the absence of any commercial or financial relationships that could be construed as a potential conflict of interest.
